# 1448. Characterizing Monkeypox in a Secondary Hospital in Madrid. Is HIV Playing a Role?

**DOI:** 10.1093/ofid/ofad500.1285

**Published:** 2023-11-27

**Authors:** Samuel Estévez Alonso, Ana Simón Gozalbo, Marta Vara González, María Gamo Guerrero, Samuel Manzano Varela, Jesús Troya García, Ekaterina Botezat, Eva Jiménez González de Buitrago, Roberto Pedrero Tomé, Maria Ángeles Martin Diaz, Pablo de la Cueva Dobao, Beatriz Fernández Gómez, Elena Palma Huertas, Elisa Fernández Vidal, David Esteban Brown Lavalle

**Affiliations:** Hospital Universitario Infanta Leonor, Madrid, Madrid, Spain; Hospital Universitario Infanta Leonor, Madrid, Madrid, Spain; Hospital Universitario Infanta Leonor, Madrid, Madrid, Spain; Hospital Universitario Infanta Leonor, Madrid, Madrid, Spain; Hospital Universitario Infanta Leonor, Madrid, Madrid, Spain; Hospital Universitario Infanta Leonor, Madrid, Madrid, Spain; Hospital Universitario Infanta Leonor, Madrid, Madrid, Spain; Hospital Universitario Infanta Leonor, Madrid, Madrid, Spain; Hospital Universitario Infanta Leonor, Madrid, Madrid, Spain; Hospital Universitario Infanta Leonor, Madrid, Madrid, Spain; Hospital Universitario Infanta Leonor, Madrid, Madrid, Spain; Hospital Universitario Infanta Leonor, Madrid, Madrid, Spain; Hospital Universitario Infanta Leonor, Madrid, Madrid, Spain; Hospital Universitario Infanta Leonor, Madrid, Madrid, Spain; Hospital Universitario Infanta Leonor, Madrid, Madrid, Spain

## Abstract

**Background:**

Monkeypox (MPOX) is a zoonotic viral disease, endemic in some Central and West African countries. However, in May 2022, cases began to be reported in non-endemic countries, demonstrating community transmission. Since the beginning of the outbreak, different epidemiological and clinical behavior have been observed.

**Methods:**

We conducted an observational study at a secondary hospital in Madrid, Spain, to characterize the MPOX suspected and confirmed cases epidemiologically and clinically. Besides the general descriptive analysis, we compared data between MPOX-confirmed cases vs MPOX-negative cases, between patients living with HIV (PLWHIV) and HIV-negative patients, and between patients vaccinated against smallpox vs unvaccinated.

**Results:**

The study comprised 133 patients with MPOX suspicion, of which 100 were confirmed. Regarding positive cases, 71.0% were PLWHIV, and 99.0% were men with a mean age of 33. Sexual behavior in the previous year included 97.6% reporting having sex with men (MSM), 53.6% using apps for sexual encounters, 22.9% practicing "chemsex”, and 16.7% with sexual intercourse in saunas. Other epidemiological data are summarized in Table 1. In the clinical aspect, the presence of inguinal lymphadenopathy was significantly higher in positive MPOX cases (54.0% vs 12.1%, p< 0.001), as the involvement of genital and perianal area (57.0% vs 27.3% and 17.0% vs 1.0%, p= 0.006 and p= 0.082 respectively). In addition, of them, 45.0% presented pustules as the most common skin lesions, and the genital area is the most frequently affected (57.0%). In PLWHIV, only 6.9% had a detectable viral load, and the mean CD4 count was 607.0/mm3. Compared to HIV-negative cases they had a trend towards greater involvement of the perianal area (3.6% vs 22.5%, p=0.05). This is more remarkable if PLWHIV had poor control criteria (CD4 count < 500 cells/mm^3^), with perianal involvement in this group at 30.3% vs 0.0% if optimal HIV control. Other clinical results are summarized in Table 2 and Table 3.

**Figure:**
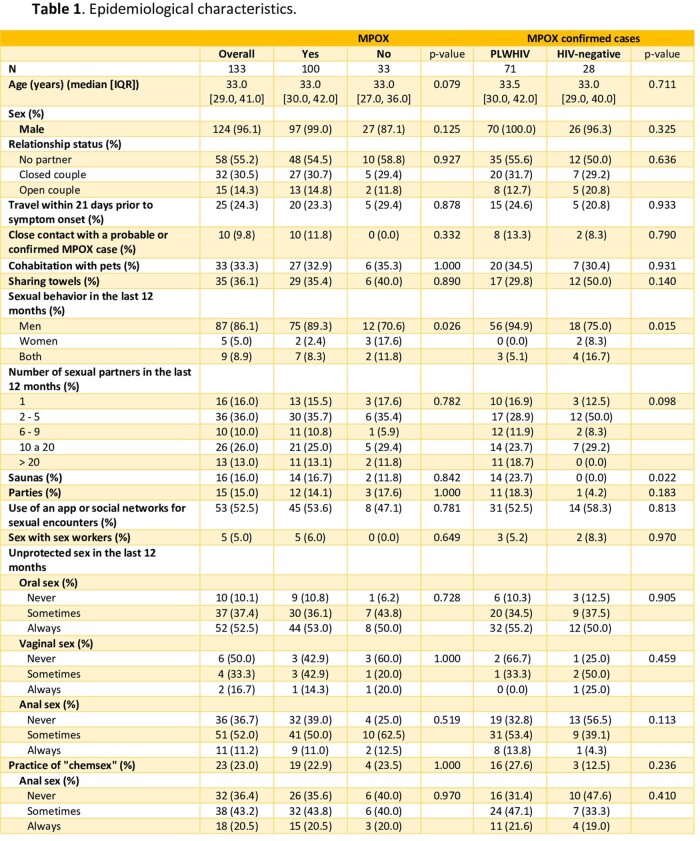

**Figure:**
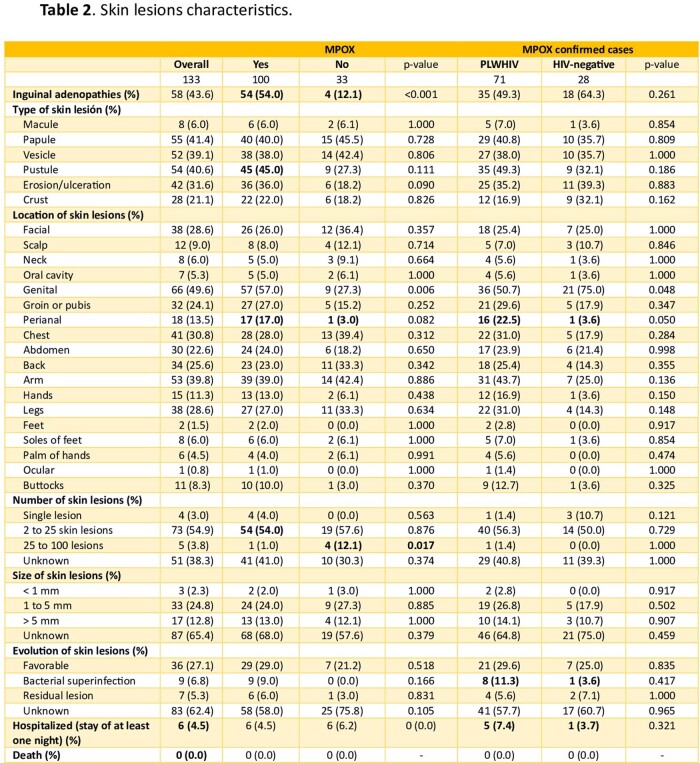

**Figure:**
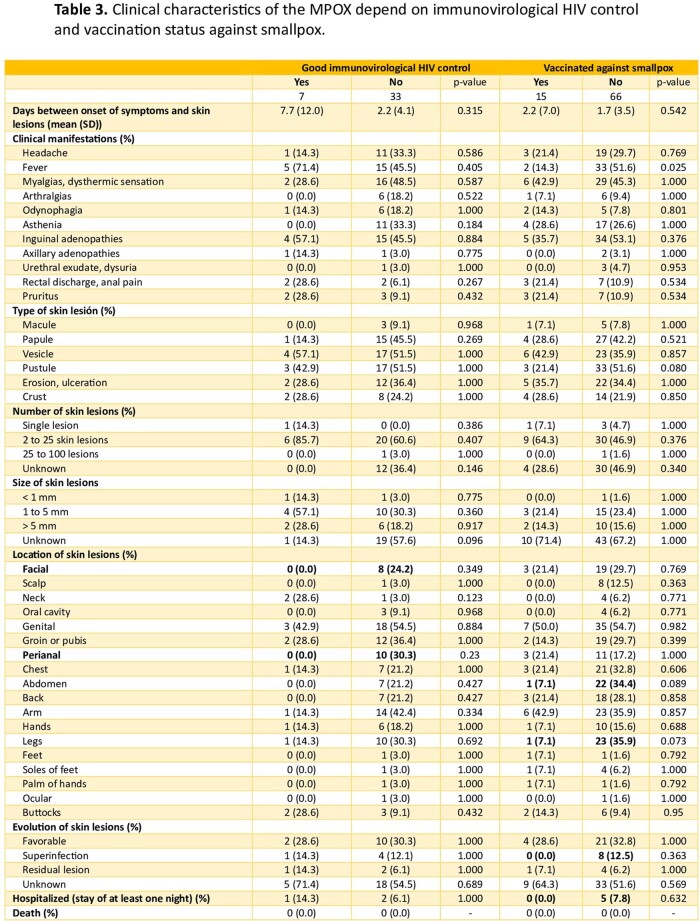

**Conclusion:**

The recent MPOX outbreak was epidemiologically related to sexual intercourses in MSM with no significant differences observed in the disease course between PLWHIV and HIV-negative cases, except for a greater tendency to appearance of perianal lesions.

**Disclosures:**

**All Authors**: No reported disclosures

